# Comparative Genomics and Virulence Mechanisms to Identify Genes Related to Mucin O‐Glycan Degradation and Pathogenicity in a Potentially Multidrug‐Resistant *Clostridium tertium* Strain

**DOI:** 10.1002/mbo3.70169

**Published:** 2025-11-24

**Authors:** Seonghun Kim, Ji Young Kang, Jung‐Sook Lee

**Affiliations:** ^1^ Jeonbuk Branch Institute Korea Research Institute of Bioscience and Biotechnology Jeongeup Korea; ^2^ Department of Biosystems and Bioengineering, KRIBB School of Biotechnology University of Science and Technology (UST) Daejeon Korea; ^3^ Korean Collection for Type Cultures, Korea Research, Institute of Bioscience and Biotechnology Jeongeup Korea

**Keywords:** cell wall component, *Clostridium tertium*, genome, mucin degradation, mucin‐type O‐glycans, multidrug resistance, pangenome, sialic acid catabolism, virulence factor

## Abstract

*Clostridium tertium* is a pathogenic bacterium that directly colonizes the gastrointestinal mucosa, causing inflammation and neutropenia. The virulence factors and pathogenic mechanisms of *C. tertium* are not well known. In this study, *C. tertium* HGMC01 was isolated by enrichment culture of human feces, and its whole chromosome genome was sequenced without extra plasmids. *C. tertium* HGMC01 had a larger genome and a higher gene count compared with five other *C. tertium* strains. A pangenome analysis of six strains showed that *C. tertium* HGMC01 had the highest number of unique genes and the lowest number of accessory genes clustered phylogenetically with *C. tertium* src5, a strain of animal origin. *C. tertium* HGMC01 genome showed a variety of secreted glycoside hydrolases and carbohydrate‐binding modules for mucin O‐glycan degradation and sialic acid catabolism including sialidase and sialic acid transporter. These genes strongly suggested that the strain could interact the human gut cells through recognition or adhesion to mucin glycans. Moreover, various mobile genetic elements in its genome also indicated the genetic diversity and plasticity of the strain to gain virulence factors and antibiotic/multidrug‐resistant genes potentially acquired by horizontal gene transfer for the evolution of the pathogenicity. Additionally, experiments with human embryonic kidney cells revealed that components of *C. tertium* HGMC01 cell wall may play roles as virulence factors by modulating cytokine signaling pathways dependent on Toll‐like receptors. Overall, this comparative genomic analysis provides information about how *C. tertium* strains cause disease through mucin glycan degradation, colonization, multidrug resistance, and modulation of immune responses.

## Graphical abstract

The comparative genomic analysis provides information about how *C. tertium* HGMC01 strain cause disease through mucin glycan degradation, colonization, multidrug resistance, and modulation of immune responses. Moreover, this analysis data suggest that this strain interacts with host cells by degrading mucin in the gut, potentially through recognition or adhesion to glycoconjugates of mucin‐type O‐glycans. Cytotoxic effects and TLR 2/4‐mediated immune response in host cells revealed that glycoconjugates in the cell wall of *C. tertium* HGMC01 are additional virulence factors.

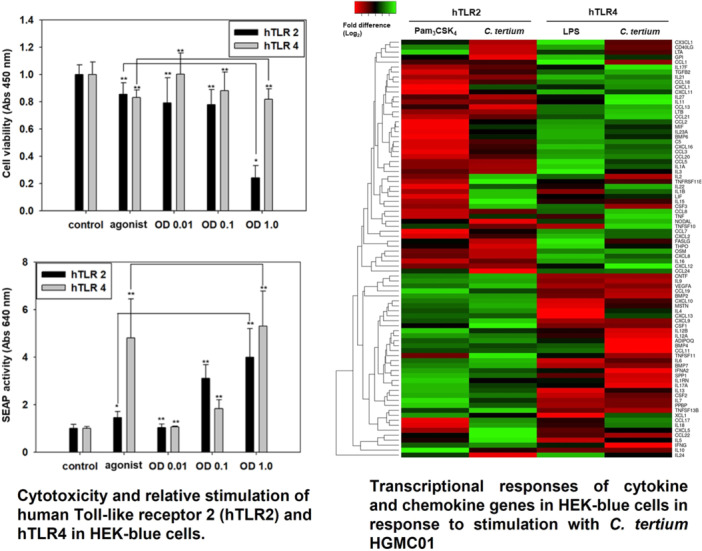

## 1 Introduction

The human gut harbors a diverse bacterial ecosystem with symbiotic relationships between the microbiome and the host. The intestinal microbiota is predominantly composed of bacteria from three phyla, namely, *Firmicutes*, *Bacteroidetes*, and *Actinobacteria* (Kho and Lal 2018). Although bacterial communities in the gut include pathogenic and nonpathogenic microorganisms, the gut microbiota play a role in protecting the host against enteric bacterial infection (Rolhion and Chassaing 2016; Kho and Lal 2018). Nonpathogenic microbes dominantly occupy the gut niche and suppress the overgrowth and colonization of potentially pathogenic microbes, or pathobionts. These interactions between microbiota and the host maintain intestinal homeostasis (Bäumler and Sperandio 2016; Rolhion and Chassaing 2016; Kho and Lal 2018). Perturbation of gut microbiota by endogenous and exogenous factors such as antibiotic treatment, diet, or inflammation leads to overgrowth of pathobionts that can drive mucosal tissue damage or chronic disease (Kho and Lal 2018).

*Clostridium* is a genus of gram‐positive, rod‐shaped, spore‐forming, obligate anaerobes belonging to the phylum Firmicutes. Most *Clostridium* strains are either predominantly commensal or pathogenic colonizers of the human intestine that can utilize various nonhost‐digestible polysaccharides to produce short‐chain fatty acids, mainly acetic acid, propionic acid, and butyric acid (Guo et al. 2020). These bacterial metabolites maintain low pH conditions in the gut, which promotes the growth of beneficial bacteria such as *Lactobacillus* and *Bifidobacteria* and stimulates mucin secretion by the host (Kho and Lal 2018; Guo et al. 2020). Nevertheless, several pathogenic species such as *Clostridium difficile*, *Clostridium perfringens*, and *Clostridium botulinum* are known to harbor a variety of secreted toxins that induce dysfunctions and disorders in the intestine by causing aberrant inflammatory responses and host cell apoptosis (Vedantam et al. 2012; Shen et al. 2019; Guo et al. 2020). *C. difficile* is well known to cause inflammatory bowel disease through its two toxins, toxins A and B, and can damage the cytoskeleton and colonic epithelial barrier integrity even after antibiotic treatment because of its capacity to develop multidrug resistance (Vedantam et al. 2012).

*Clostridium tertium* is rarely considered a human pathogen because of its low virulence. Nevertheless, it is capable of causing bacteremia, and several pathogenic strains have been isolated and identified from clinical samples associated with diseases including pneumonia, meningitis, septic arthritis, enterocolitis, and hepatic abscess (Thaler et al. 1986; Johnson and Tenover 1988; Rampling 1988; Coleman et al. 1993; Steyaert et al. 1999; Miller et al. 2001; Tappe et al. 2005; Bonda et al. 2022; Justesen et al. 2022; Kim et al. 2023). Besides, the clinical isolated strains showed antimicrobial resistance patterns (Thaler et al. 1986; Steyaert et al. 1999; Alvarado‐Rodríguez and Quesada‐Gómez 2025). Despite these clinical reports, its infection mechanism and pathogenicity are still unclarified. Recently, it has been reported that *C. tertium* can directly colonize the gastrointestinal tract of animals and humans (Kiu et al. 2017). As a pathogen, *C. tertium* might be able to infect a host by a sialic acid‐mediated mechanism or by adhesion, similar to other Clostridial species (Lewis and Lewis 2012). Genes encoding sialidase, a potential virulence factor, and enzymes involved in sialic acid catabolism were partially identified in *C. tertium*, and the biochemical properties of the sialidase were characterized (Grobe et al. 1998); however, the virulence factors and pathogenicity mediated by host glycoconjugates are not yet known.

Additionally, strains of mucin‐degrading bacteria, including *C. tertium* WC0709, were isolated from human fecal samples by enrichment culture with mucin as a sole carbon source (Raimondi et al. 2021). Genomic and functional analyses of the isolated strains revealed several glycosyl hydrolases and showed that the catabolism of mucin‐hydrolyzed monosaccharides was associated with antibiotic resistance, biofilm formation, and tolerance to oxygen and temperature (Candeliere et al. 2024). However, analyses of clinical *C. tertium* strains showed dynamic genomic variations due to horizontal gene transfer (HGT) or adaptive evolution, resulting in gain or loss of toxins, virulence factors, and genes involved in antibiotic resistance, sporulation/germination, and mucin glycan catabolism (Brouwer et al. 2013; Stecher et al. 2013; Juhas 2015; Kiu et al. 2017; Muñoz et al. 2019). Moreover, bacterial cell wall polysaccharides can be potential virulence factors, as in the case of *C. difficile* glycopolymer, but their biological activities in *C. tertium* strains have not yet been reported (Chu et al. 2016).

In this study, *C. tertium* HGMC01 isolated from an enrichment culture of human feces was investigated for its potential pathogenicity and virulence. The whole genome of *C. tertium* HGMC01 was completely sequenced and compared to five previously identified *C. tertium* strains. The analysis was designed to identify genes encoding enzymes for mucin degradation and catabolism of carbohydrates released from host mucin‐type O‐glycans, which would identify this strain as a mucin degrader in the human gut intestinal niche. In addition, various mobile genetic elements, virulence factors, and antibiotic‐/multidrug‐resistance genes were analyzed for strain‐specific pathogenicity. Experiments with fixed whole *C. tertium* HGMC01 cells and human embryonic kidney (HEK) cells stably expressing toll‐like receptor 2 (TLR2) or TLR4 were conducted to test the ability of bacterial cell wall components to induce cytotoxicity or TLR‐dependent cytokine signaling. The results suggest that *C. tertium* HGMC01 is a mucin O‐glycan degrader harboring various virulence factors with high potential for multidrug resistance.

## 2 Materials and Methods

### 2.1 Bacteria Isolation and Molecular Identification

*C. tertium* HGMC01 was identified from bacteria pools isolated from in vitro gut microbiome consortia (Kim et al. 2024). In vitro gut microbiome consortia were serially diluted, spread on brain–heart infusion (BHI) agar plates, and incubated at 37°C under anaerobic condition. Single colonies were picked randomly and cultivated in BHI broth. Cells were harvested from the pure cultures, resuspended in 30% (v/v) glycerol, and maintained at –80°C for strain identification. The isolated strains were identified by analysis of the rDNA locus in genomic DNA extracted from cultivated cells using the AccuPrep Genomic DNA Extraction Kit (Bioneer, Daejeon, Korea). The 16S rDNA gene was amplified by PCR using universal primers 27F (5′‐AGAGTTTGATCCTGGCTCAG‐3′) and 1492R (5′‐TACGGYTACCTTGTTACGACTT‐3′) with the genomic DNA as the template. PCR products were purified using the QIAquick Gel Extraction Kit (Qiagen, Valencia, CA, USA). Purified DNA fragments were cloned into the pGEM‐T Easy vector (Promega, Madison, WI, USA) and sequenced. The nucleotide sequences of the 16S rDNA amplicon were deposited into the GenBank database under accession number PQ538582‐PQ538587. Sequence data were compared to homologous DNA sequences of other *C. tertium* strains in the NCBI and GenBank databases to confirm the identity of *C. tertium* HGMC01.

### 2.2 Genome Sequencing, De Novo Assembly, and Annotation

Genomic DNA was extracted from *C. tertium* cultivated in BHI broth using the AccuPrep Genomic DNA Extraction Kit (Bioneer, Daejeon, Korea) according to the manufacturer's instructions. A whole‐genome sequencing library was constructed using the genomic DNA and a DNA template prep kit (v3.0) for PacBio RS II according to the manufacturer's protocols. Genomic sequencing was performed at Macrogen (Seoul, Korea) using single‐molecule real‐time (SMRT) cells on the PacBio RS II platform (Pacific Biosciences of California, Menlo Park, CA, USA). Raw read data were analyzed with Jellyfish (v2.2.10) for genome size prediction, genome coverage confirmation, and repeat sequence ratio calculation and with GenomeScope to determine the k‐mer count distribution. The PacBio reads were assembled using Canu (v1.3) with default settings. Subreads were assembled using the hierarchical genome assembly process (HGAP v3.0) and SMRT Portal (v2.3) de novo assembler with default options. The merged contigs were checked, circularized, reordered, and polished using Pilon (v1.21) to further improve the assembly and validation. A benchmarking universal single‐copy ortholog (BUSCO, v3.0) analysis was performed to assess the completeness of the genome assembly, with bacteria_odb9 as the default value (Waterhouse et al. 2018). The completely assembled genome was used with the Annotation NCBI Prokaryotic Genome Annotation Pipeline (PGAP) v.6.4 (Tatusova et al. 2016) for gene predictions and functional annotations. Further annotations for predicted protein sequence sets were performed using Inter‐ProScan v5.30‐69.0 and psiblast v2.4.0 with EggNOG database v4.5 and COG database v2014 (Camacho et al. 2009; Jones et al. 2014; Huerta‐Cepas et al. 2017; Galperin et al. 2021).

### 2.3 Prediction of Carbohydrate‐Active Enzymes (CAZymes)

To predict CAZymes, the functional amino acid sequences of *C. tertium* HGMC01 were annotated using (1) the HMMER v3.3.2 package in the dbCAN2 server (https://bcb.unl.edu/dbCAN2/), running against Pfam Hidden Markov Models (HMMs), and 2) the EggNOG database v5.0 (http://eggnog5.embl.de/) with the web‐based eggNOG‐mapper v2 (http://eggnog-mapper.embl.de/).

### 2.4 Comparative Pangenome Analysis of *C. tertium* Strains

A comparative pangenome analysis was performed using the complete genomes of *C. tertium* HGMC01 and five other previously sequenced *C. tertium* strains: *C. tertium* Gcol_A2 (NCBI accession number GCA_003284625.1), *C. tertium* Gcol_A43 (GCA_003284645.1), *C. tertium* LH009 (GAC_900217175.1), *C. tertium* src5 (GCA_900205935.1), and *C. tertium* MGYG‐HGUT‐01328 (GCF_902373955.1) (Muñoz et al. 2019). The gene annotation files (.gff3) of the strains were analyzed using Roary v3.13.0 with BLASTP, with the default cutoff of 95% sequence identity in the Roary pipeline (Page et al. 2015). Functional enrichment of ortholog clusters determined using the PGAP program was used to classify clusters of orthologous groups (COGs) described in the genome annotation. After running the Roary pipeline, all of the genes in the pangenome were classified as belonging to either the core genome (> 99%) or the shell genome (< 95%–≥ 15%; Supporting Data S1). The output files from Roary were used to analyze and visualize the core and shell genomes of the *C. tertium* strains with the roary_plots.py Python script (https://sanger-pathogens.github.io/Roary/).

### 2.5 Prediction of Mobile Genetic Elements, Virulence Factors, and Antibiotic Resistance Genes

Mobile genetic elements were analyzed using BLASTP searches against the mobileOG‐db database (https://mobileogdb.flsi.cloud.vt.edu/) (Brown et al. 2022), the Phigaro Python package (http://dmk-brain.ecn.uiowa.edu/pVOGs/) (Starikova et al. 2020), and IS finder (https://isfinder.biotoul.fr/) (Siguier 2006). Virulence factor genes were identified using PathogenFinder (https://cge.food.dtu.dk/services/PathogenFinder/) (Cosentino et al. 2013) and by performing amino acid sequence alignments against a virulence factor database (http://www.mgc.ac.cn/VFs/) (Liu et al. 2022). Antibiotic‐/multidrug‐resistance genes were predicted by BLAST searches against the comprehensive antibiotic resistance database (CARD) reference sequences (https://card.mcmaster.ca/analyze) (Alcock et al. 2023), using alignments with pass_bitscore > 600 and best_hit_bitscore > 50. The abundances of these genes in the *C. tertium* HGMC01 genome were visualized using Proksee (https://proksee.ca/) (Grant et al. 2023).

### 2.6 HEK‐Blue Cell Cultivation, TLR Assay, and Viability Testing

For preparation of fixed bacteria, *C. tertium* HGMC01 was cultivated in BHI broth until the OD_600_ reached 2–2.5. The cells were then harvested, washed with phosphate‐buffered saline (PBS) three times, resuspended in 0.1M phosphate buffer (pH 7.2) containing 2% (v/v) glutaraldehyde, and incubated at 4°C overnight. After overnight fixation, the cells were harvested and washed with a 50%, 70%, 85%, 95%, 100% (v/v) ethanol series. The fixed bacterial cells were finally suspended in PBS for whole‐cell treatment assays.

HEK‐blue hTLR2 or hTLR4 cells (InvivoGen, San Diego, CA, USA) were cultivated at 37°C in a humidified incubator with 5% CO_2_ in DMEM supplemented with 10% fetal bovine serum, penicillin (100 units/mL), streptomycin (100 μg/mL), normocin (100 μg/mL), and 2 mM L‐glutamine. The cultivated cells were then seeded in 96‐well plates at a density of 1 × 10^5^ cells/well and cultured overnight. After overnight culture, the cells were stimulated with different concentrations of fixed *C. tertium* cells (OD_600_ = 0.01, 0.1, or 1), Pam_3_CSK_4_ (5 ng/mL) as a positive control for TLR2, or lipopolysaccharide (LPS; *Escherichia coli* O55B5, 50 ng/mL) as a positive control for TLR4. After 16 h of incubation at 37°C, secreted embryonic alkaline phosphatase (SEAP) activity was measured using HEK‐blue detection solution (InvivoGen), following the manufacturer's protocol. For cytotoxicity assays, HEK‐blue cells cultivated in 96‐well plates were treated with whole *C. tertium* cells under the same conditions described above, and cell viability was assessed using cell counting kit‐8 (Dojindo Laboratories, Kumamoto, Japan), following our previous procedures (Kim 2023). Data were analyzed by one‐way or two‐way analysis of variance (ANOVA) using Sigma Plot 14.5 (Systat Software, Warrenton, VA, USA). A *p*‐value < 0.05 was considered to be statistically significant. Differences between groups were analyzed by Bonferroni's multiple range test. The result is resented as the mean values with the standard errors for each variable of five independent replicates (*n* = 5).

### 2.7 Quantitative Reverse‐Transcription PCR (RT‐qPCR)

Total RNAs were isolated from HEK‐blue hTLR2 or hTLR4 cells treated with fixed whole *C. tertium* cells (OD_600_ = 0.1) using an RNeasy Mini Kit (QUIAGEN, Hilden, Germany) according to the manufacturer's protocol. The quality and quantity of extracted total RNA were measured using Eppendorf BioSpectrometer fluorescence. The RNA was reverse‐transcribed using oligo dT primers and SuperScript II reverse transcriptase (Thermofisher, Waltham, MA, USA). The transcript level of each gene was quantitatively determined by qPCR using a BioRad Real‐Time PCR System (BioRad, Foster City, CA, USA) with SYBR Green PCR master mix (Thermofisher). Amplification by qPCR was performed using an AccuTarget Human qPCR screening kit with cytokine‐ and chemokine‐specific primers (Bioneer, Daejeon, Korea) under the recommended reaction conditions. The threshold cycle (*Ct*) value of each gene target was normalized to that of the endogenous GAPDH or actin genes (Δ*Ct* value), and the relative differences in the expression levels of each gene between groups (ΔΔ*Ct*) were determined as fold change (2^−ΔΔ*Ct*^) values. The expression differences of each gene were visualized using Heatmapper (http://heatmapper.ca/).

## 3 Results

### 3.1 Genome Analysis of *C. tertium* HGMC01

A total of 159,434 subreads (N_50_ value = 12,798) with an average of 9035 bp per subread (total 1,440,531,167 bp) were generated for *C. tertium* HGMC01 by the PacBio sequencer. After de novo assembly, the complete genome of *C. tertium* HGMC01 consisted of one circular chromosome of 4,076,529 bp with 28.05% GC content (Table 1). A comparison of the assembly statistics showed that *C. tertium* HGMC01 had similar GC content but significantly different genome size and number of coding genes compared with the other five *C. tertium* strains, which was reported in previous study (Muñoz et al. 2019). The BUSCO analysis detected 143 complete and single‐copy BUSCOs (96.62%) in the *C. tertium* HGMC01 genome, with five missing BUSCOs (3.38%). The completely assembled genome contained 3705 coding genes (functionally assigned as 3627 protein‐coding genes and 78 hypothetical protein genes) and 113 noncoding genes, including 86 transfer RNAs (tRNAs) and 30 ribosomal RNAs (rRNAs).

**Table 1:** Comparison of genomic features of *Clostridium tertium* strains.

Strain name	*C. tertium*					
	HGMC01	Gcol.A2	Gcol.A43	src5	LH009	MGYG‐HGUT‐01328
Total length (bp)	4,076,529	3,897,924	3,801,844	3,902,863	3,970,462	3,813,122
No. of contigs	1	60	55	68	49	5
N50 (bp)	12,798	281,242	178,423	108,109	230,975	146,762
G + C content (%)	28.05	28.12	29.05	27.91	27.78	27.80
No. of genes	3821	3743	3591	3743	3886	3629
CDS	3705	3585	3440	3621	3723	3486
tRNA	86	83	82	60	88	74
rRNA	30	13	11	16	0	18
OrthoANI value (%)	100	98.51	98.47	98.47	98.47	98.29
NCBI accession number	SAMN44517393	GCA_003284625.1	GCA_003284645.1	GCA_900205935.1	GCA_900217175.1	GCA_902373955.1
Reference	This study	Muñoz et al. (2019)	Muñoz et al. (2019)	Muñoz et al. (2019)	Kiu et al. (2017)	EMG whole genome data^a^

^a^ EMBL‐EBI metagenomics team, human gut genome, EMG, The European Bioinformatics Institute (EMBL‐EBI), Wellcome Genome Campus, CB10 1 SD, United Kingdom.

### 3.2 Average Nucleotide Identity (ANI)

To evaluate phylogenetic relationships, the 16S rDNA of *C. tertium* HGMC01 was compared to that of 71 other sequenced *C. tertium* strains in GenBank. The results revealed highly conserved relationships and tight clustering among *C. tertium* species (data not shown). *C. tertium* HGMC01 showed high sequence identity with *C. tertium* strains Gcol.A2 (GCA_003284625.1), Gcol_A43 (GCA_003284645.1), LH009 (GAC_900217175.1), src5 (GCA_900205935.1), and MGYG‐HGUT‐01328 (GCF_902373955.1). Therefore, further comparisons were made using *C. tertium* HGMC01 and these five strains, which were previously shown to share > 95.0 average nucleotide identity with each other (Muñoz et al. 2019). The average nucleotide identity between *C. tertium* HGMC01 and *C. tertium* strains Gcol.A2, Gcol.A43, src5, LH009, and MGYG‐HGUT‐01328 was 98.51%, 98.47%, 98.47%, 98.47%, and 98.29%, respectively, although total genome length and genome coverage differed among the strains (Table 1). These ANI data clearly showed that *C. tertium* HGMC01 belongs to the *C. tertium* species group rather than some other group within the *Clostridium* genus.

### 3.3 Pangenome Analysis of *C. tertium* Strains

**Figure 1:**
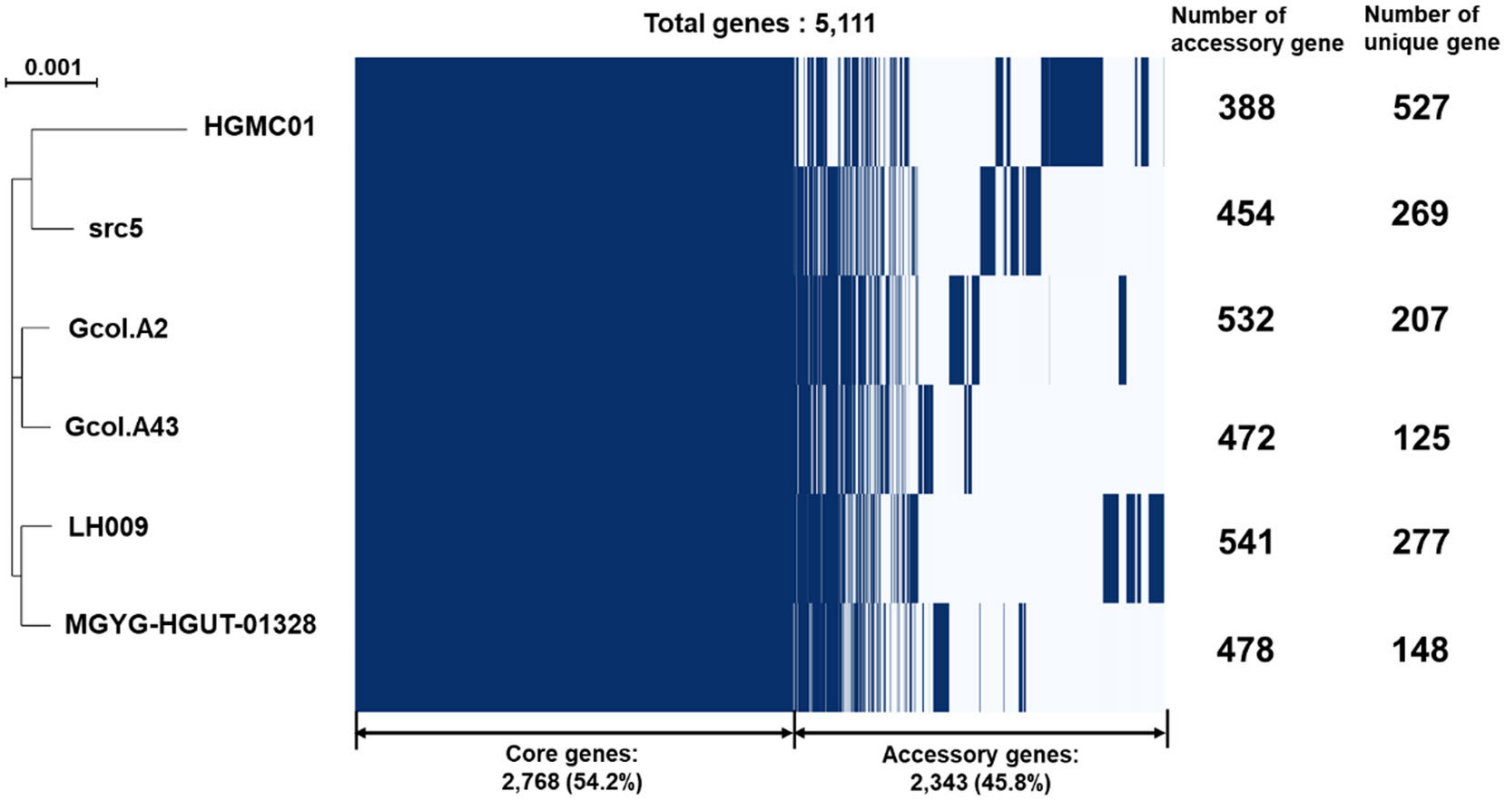
Pangenome analysis of *Clostridium tertium* HGMC01 and five other strains of the same species. Pairs of genes with > 95% identical amino acid sequences were defined as belonging to an orthologous gene family. The numbers of genes classified as core genes (99% ≤ strains ≤ 100%) and shell genes (15% ≤ strains < 95%) were 2768 and 2343, respectively. A total of 5111 genes were included in the pangenome analysis. A phylogenetic tree was constructed based on the core genes (left panel). The numbers of accessory and unique genes in each *C. tertium* genome are shown (right panel).

To identify unique genes in the *C. tertium* genome, the pangenome of *C. tertium* HGMC01 and five closely related strains of the same species were analyzed (Supporting Data S1). The pangenome analysis revealed a total of 5111 *C. tertium* genes, which included 2768 (54.2%) core genes and 2343 (45.8%) accessory genes (Figure 1). The accessory genes included 590 strain‐specific genes (282 genes in five strains, 170 genes in four strains, 99 genes in three strains, and 239 genes in two strains) and 1753 unique genes. *C. tertium* HGMC01 showed the highest number of unique genes (527) and the lowest number of strain‐specific genes (388) among the *C. tertium* strains. A phylogenetic tree constructed using the core genes showed that *C. tertium* HGMC01 clustered separately with *C. tertium* src5 (Figure 1), an isolate of animal origin rather than human origin (Kiu et al. 2017; Muñoz et al. 2019).

### 3.4 Functional Annotation

**Figure 2:**
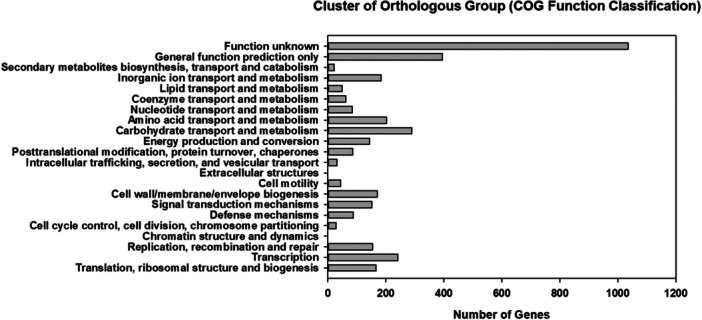
Cluster of orthologous groups (COG) functional annotation of genes in the *Clostridium tertium* HGMC01 genome. Each bar indicates the number of genes assigned to a COG functional category.

To assign the functionality of the core and accessory protein‐coding genes, gene ontology was analyzed using the COG and EggNOG databases. Of the 3705 coding genes in *C. tertium* HGMC01, 3590 (96%) were assigned to 23 functional categories (Figure 2). The highest number of genes (1035; 28.54%) were categorized as “function unknown.” The second highest number of genes (395; 10.89%) were grouped as “general function prediction only.” Other genes were dominantly functionally categorized as “carbohydrate transport and metabolism” (7.97%), “transcription” (6.65%), “amino acid transport and metabolism” (5.57%), “inorganic ion transport and metabolism” (5.05%), “cell wall/membrane/envelope biogenesis” (4.69%), “replication, recombination and repair” (4.25%), “signal transduction mechanisms” (4.19%), and “energy production and conversion” (3.97%). The rest of the genes were categorized in minority groups as “defense mechanisms” (2.43%), “posttranslational modification, protein turnover, chaperones” (2.37%), “nucleotide transport and metabolism” (2.32%), “coenzyme transport and metabolism” (1.71%), “lipid transport and metabolism” (1.35%), “cell motility” (1.21%), “intracellular trafficking, secretion, and vesicular transport” (0.86), “cell cycle control, cell division, chromosome partitioning” (0.77%), and “secondary metabolites biosynthesis, transport and catabolism” (0.61%).

### 3.5 Identification of CAZymes and Potential Mucin Degradation Enzymes

To investigate the potential for enzymatic digestion of polysaccharides, CAZymes were analyzed in the *C. tertium* HGMC01 genome. The annotation of CAZymes was performed using the CAZy database with the dbCAN annotation algorithm. CAZymes were classified as belonging to one of six families: glycoside hydrolases (GHs), glycosyltransferases (GTs), carbohydrate‐binding modules (CBMs), auxiliary activities (AAs), carbohydrate esterases (CEs), and polysaccharide lyases (PLs). Out of 3705 coding genes, a total of 209 (5.6%) CAZymes were identified with a cutoff *E*‐value < 1e–5, including 80 GHs (38.3%), 63 GTs (30.1%), 5 PLs (2.4%), 22 CEs (10.5%), 9 AAs (4.3%), and 30 CBMs (14.4%; Table 2). Only 28 (13.4%) proteins in the genome were identified as secreted CAZymes with signal sequences, however. The secreted CAZymes included 20 GHs (67.9% of the total secreted proteins in the genome), 5 CEs (17.9%), 1 PL (3.6%), and 2 CBMs (7.1%). No secreted AAs or GTs were identified. These results clearly demonstrate that GHs were the predominant enzymes among the predicted extracellular proteins for polysaccharide degradation.

**Table 2:** Carbohydrate‐active enzymes and associated modules annotation.

Classification	Total number	Superfamily number	Secreted enzyme
Glycoside hydrolases (GHs)	80	48	20
Glycosyl transferases (GTs)	63	18	0
Polysaccharide lyases (PLs)	5	4	1
Carbohydrate esterases (CEs)	22	8	5
Auxiliary activities (AAs)	9	2	0
Carbohydrate‐binding modules (CBMs)	30	7	2

**Figure 3:**
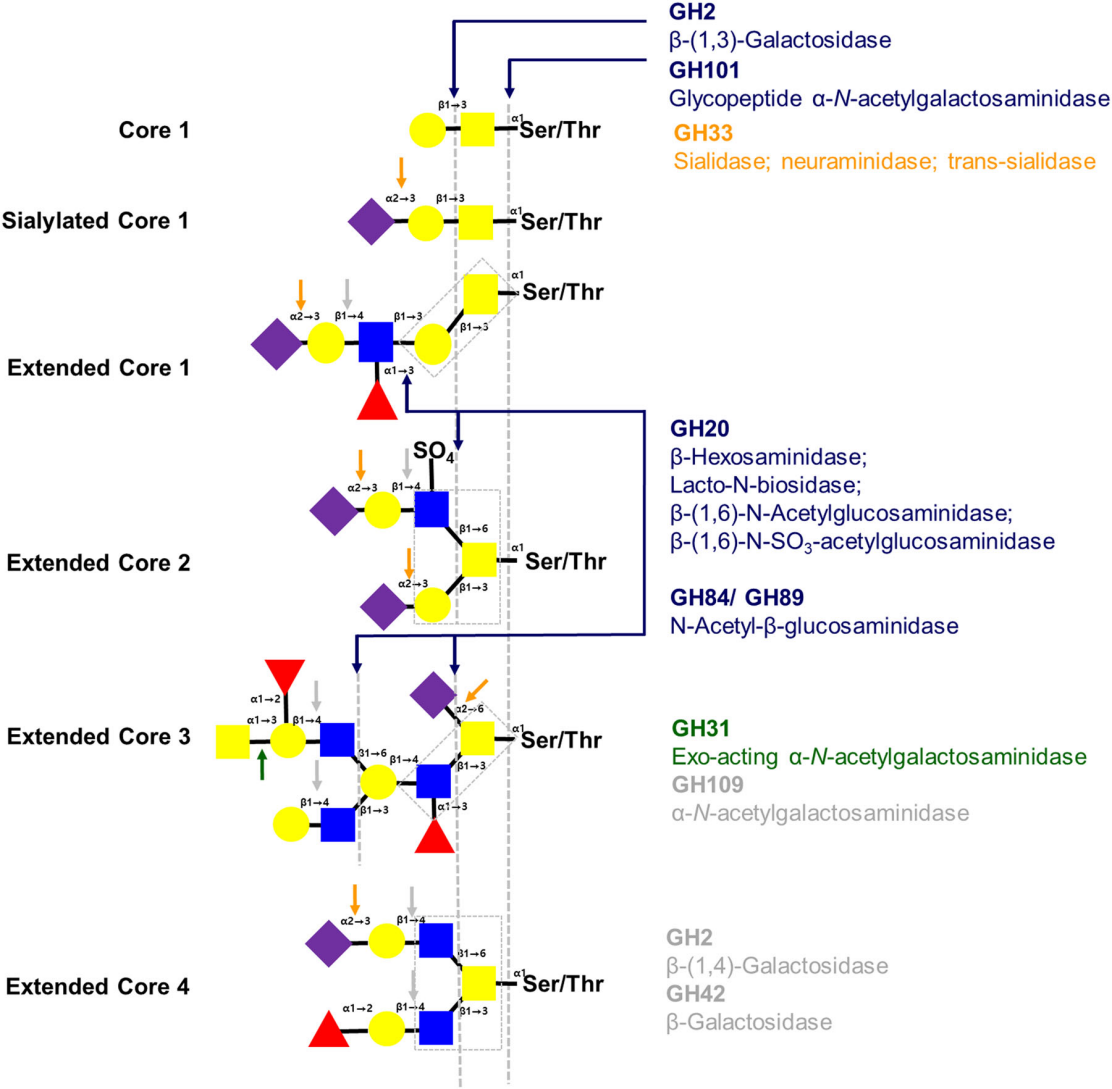
Schematic presentation of the putative mucin‐type O‐glycan degrading CAZymes annotated in *Clostridium tertium* HGMC01 genome. The secreted and nonsecreted GHs were marked with bold colorful and gray letters, respectively. The arrows indicated the targeting hydrolysis linkages in the mucin glycan structures.

The identified GHs were analyzed for predicted ability to degrade mucin glycoprotein in the gut. Twenty‐two putative mucin‐type O‐glycan‐degrading CAZymes were identified (Table 3); however, only 10 secreted CAZymes were predicted to degrade and bind glycan structures (Figure 3). Among these was a GH33 sialidase (C‐tertium_1_00181), which cleaves terminal sialic acid residues to degrade O‐glycans. For subsequent extracellular degradation of glycan chains, the genome contained a GH2 β‐galactosidase (C‐tertium_1_00200); GH20 (C‐tertium_1_00149), GH84 (C‐tertium_1_00199, C‐tertium_1_00574, and C‐tertium_1_00575), and GH89 (C‐tertium_1_00293) *N*‐acetyl‐glucosaminidases; and GH31 (C‐tertium_1_03782) and GH101 (C‐tertium_1_00119) *N*‐acetyl‐galactosaminidases. In addition, the genome contained a secreted protein consisting of CBM51/CBM32 to digest the galactose moiety bound by blood group A/B‐antigen. A GH112 lacto‐*N*‐biose phosphorylase (C‐tertium_1_03179) was detected, but this protein did not include a signal peptide sequence. Proteins to degrade fucose residues (GH29 or GH95) or lacto‐N‐biosidase (GH136) were not detected.

**Table 3:** Putative mucin‐type O‐glycan degrading CAZymes.

Locus tag	Signal peptide (Amino acids)	CAZy family	CBM	Putative enzyme activity (Substrate)
C‐tertium_1_00200	Y (1–30)	GH2	CBM71	β‐1,3‐galactosidase (Gal β‐(1,3)‐GlcNAc); β‐1,4‐galactosidase (Gal β‐(1,4)‐GlcNAc);
C‐tertium_1_00215	N	GH2		
C‐tertium_1_00442	N	GH2		
C‐tertium_1_01443	N	GH2		
C‐tertium_1_03758	N	GH2		
C‐tertium_1_00074	N	GH20		β‐hexosaminidase; Lacto‐*N*‐biosidase; β‐(1,6)‐*N*‐acetecylglucosaminidase; β‐(1,6)‐*N*‐SO_3_‐acetecylglucosaminidase
C‐tertium_1_00149	Y (1–31)	GH20	CBM32	
C‐tertium_1_02228	N	GH20		
C‐tertium_1_00112	N	GH31		Exo‐acting protein‐α‐*N*‐acetyl galactosaminidase
C‐tertium_1_01303	N	GH31		
C‐tertium_1_03484	N	GH31		
C‐tertium_1_03782	Y (1–32)	GH31	CBM32	
C‐tertium_1_00181	Y (1–37)	GH33	CBM40	Sialidase; neuraminidase; trans‐sialidase (Neu5Ac‐α(2,3(6)‐Gal/GalNAc)
C‐tertium_1_00320	N	GH42		β‐galactosidase; α‐L‐arabinopyranosidase
C‐tertium_1_00199	Y (1–31)	GH84	CBM32	*N*‐acetyl‐β‐glucosaminidase; Hyaluronidase; 3‐O‐(GlcNAc)‐L‐Ser/Thr β‐*N*‐acetylglucosaminidase
C‐tertium_1_00574	Y (1–36)	GH84		
C‐tertium_1_00575	Y (1–30)	GH84	CBM32	
C‐tertium_1_00293	Y (1–31)	GH89	CBM32	α‐*N*‐acetylglucosaminidase
C‐tertium_1_00119	Y (1–35)	GH101		Glycopeptide α‐*N*‐acetylgalactosaminidase
C‐tertium_1_00267	N	GH109		α‐*N*‐acetylgalactosaminidase
C‐tertium_1_03179	N	GH112		β‐(1,3)‐galactosyl‐*N*‐acetylhexosamine phosphorylase
C‐tertium_1_03740	Y (1‐28)	‐‐‐	CBM51/CBM32	Galactose binding protein (blood group A/B‐antigen)

### 3.6 Sialic Acid Catabolism

Sialic acid catabolism is one determinant of virulence in commensal and pathogenic bacteria (Lewis and Lewis 2012). In the sialic acid catabolism pathway, free sialic acid cleaved from host glycoconjugates by sialidase (NanH) enters into cells via a specific transporter system and is metabolized to *N*‐acetylglucosmine‐6‐phosphate by sequential reactions catalyzed by neuraminic acid lyase (NanA), *N*‐acetyl‐mannosamine (ManNAc) kinase (NanK), and *N*‐acetyl mannosamine epimerase (NanE) (Lewis and Lewis 2012). The generated *N*‐acetylglucosmine‐6‐phosphate is further metabolized by deacetylase (NagA) and deaminase (NagB) to fructose‐6‐phosphate, which enters into the glycolysis pathway. The genes encoding the NanA/K/E enzymes for the initial three steps of free sialic acid metabolism are often clustered together in bacterial genomes (Lewis and Lewis 2012).

The *C. tertium* HGMC01 genome contained genes encoding all the enzymes needed for sialic acid catabolism (Table 4). The genome contained only one gene each for sialidase, sialic acid transporter, ManNAc kinase, and glucosamine‐6‐phosphate deaminase, whereas it contained two or three genes for each of the other enzymes in the sialic acid catabolism pathway. The genes encoding NanH (C‐tertium_1_00181) and *N*‐acetyl neuraminic acid lyase 1 (*nan*A1; C‐tertium_1_00182) were located together in a gene cluster, as were the genes encoding NanK (C‐tertium_1_02001), *N*‐acetylmannosamine‐6‐phosphate 2‐epimerase 2 coding (*nan*E2; C‐tertium_1_02002), and N‐acetyl neuraminic acid lyase 2 (*nan*A2; C‐tertium_1_02006). The other genes in the sialic acid catabolism pathway were distributed separately across the genome. In addition, the genome contained a gene encoding the major facilitator superfamily sialic acid transporter NanT (*nan*T; C‐tertium_1_03759), which transports free sialic acid into the cell. More than 100 genes encoding putative ATP binding cassette (ABC) transporters were also identified (data not shown). No genes for sialic acid biosynthesis, such as sialyltransferase, were detected in the genome.

**Table 4:** Putative sialic acid catabolism‐related genes in *C. tertium* HGMC01.

Locus tag	Genotype	Function
C‐tertium_1_00181	*nan*H	Sialidase
C‐tertium_1_03759	*nan*T	Sialic acid transporter
C‐tertium_1_00182	*nan*A1	*N*‐acetyl neuraminic acid lyase 1
C‐tertium_1_02006	*nan*A2	*N*‐acetyl neuraminic acid lyase 2
C‐tertium_1_02001	*nan*K	*N*‐acetylmannosamine (ManNAc) kinase
C‐tertium_1_00174	*nan*E1	*N*‐acetylmannosamine‐6‐phosphate 2‐epimerase 1
C‐tertium_1_02002	*nan*E2	*N*‐acetylmannosamine‐6‐phosphate 2‐epimerase 2
C‐tertium_1_03258	*nan*E3	*N*‐acetylmannosamine‐6‐phosphate 2‐epimerase 3
C‐tertium_1_02895	*nag*A1	*N*‐acetylglucosamine‐6‐phosphate deacetylase 1
C‐tertium_1_03780	*nag*A2	*N*‐acetylglucosamine‐6‐phosphate deacetylase 2
C‐tertium_1_03566	*nag*B	Glucosamine‐6‐phosphate deaminase

### 3.7 Mobile Genetic Elements

Mobile genetic elements in *Clostridium* strains provide genetic plasticity for the evolution of pathogenicity and drug resistance (Broaders et al. 2013). Moreover, diverse mobile genetic elements affect *C. tertium* genome structure and function through the insertion and deletion of large DNA segments, resulting in differences in genome size among strains. A mobileOG‐db analysis (Brown et al. 2022) predicted 120 putative mobile genetic elements in five functional categories (Supporting Data S2): Replication, recombination, or nucleic acid repair (42); phage‐specific biological processes (30); Integration and excision from one genetic locus to another (25); inter‐organism transfer (18); and element stability, transfer, or defense (7). In addition, functional annotation of the genome showed 26 putative transposases with roles in integration and excision, including 17 uncharacterized transposases, 3 retrotransposon proteins, 2 Tn7 transposases, 1 Tn10, 1 Tn916, and 1 TnFO1. These results suggest that *C. tertium* HGMC01 is capable of rapidly generating high genetic diversity via different types of mobile genetic elements.

CRISPRCasFinder (https://crisprcas.i2bc.paris-saclay.fr/CrisprCasFinder/Index) predicted two orphan CRISPR arrays without a Cas gene cluster in the *C. tertium* HGMC01 genome. The first CRISPR array (start–end positions: 605,811 bp–605,997 bp) harbored a direct repeat (DR) sequence, DR1 (AATTAATGAAATAATTCAGGCAAG), with 61.75% conserved homology and three spacers (CCAAGAAGAATGGAGAAGTGATAATGGACAATTGAT; TAGAAATACAATTGATAATTGGCAATGGATAATGGAC; CTGAATTATGCAAGAATG). The second CRISPR array (3,775,742–3,775,834 bp) harbored a DR2 sequence (CTTCCAAATTTTATATTTAGCAA) with 95.65% conserved homology and one spacer (GGCGAACTTACTCATTCGAATGCATATGAGAAGGATTTACAACAA AA). The DR1 and DR2 sequences were completely different from each other, with lengths of 24 and 23 bp, respectively.

Phigaro (Starikova et al. 2020) predicted three prophage loci (P1, P2, and P3) in the *C. tertium* HGMC01 genome, with sizes (start–end positions) of 8744 bp (987,468–996,211), 5825 bp (2,174,605–2,180,429), and 13,254 bp (2,609,748–2,623,002), respectively. The total length of the intact prophage fragments (about 27.8 kb) accounted for 0.68% of the bacterial genome. The GC content of the prophage loci was 30.27%, which was higher than that of the host genome (28.05%). When the three intact prophage sequences were aligned to the standard nucleotide sequences in the NCBI database, matches with > 80% identity to *Caudoviricetes* sp., *Bacteriophage* sp., *Siphoviridae* sp., and *Clostridium* phages were identified. Interestingly, all three prophage sequences matched *Caudoviricetes* sp. phages (Supporting Data S3), suggesting that they might have originated from those phages. Moreover, the strain alignment results showed that the three prophages in the *C. tertium* HGMC01 genome were highly prevalent and widespread in other *Clostridium* strains (Supporting Data S3).

Insertion sequences (ISs) are small, mobile genetic elements that are widely distributed in many bacterial genomes (Udaondo et al. 2022). ISfinder (https://www-is.biotoul.fr/) (Siguier 2006) predicted that *C. tertium* HGMC01 harbored a high number of ISs (*n* = 165) belonging to 27 distinct families of ISs/transposes. Among these, three transposase families (IS200/IS605, *n* = 29; IS3, *n* = 24; and IS1595, *n* = 18) were highly dominant in the genome, whereas another five families (IS4, *n* = 11; IS1182, *n* = 11; IS5, *n* = 8; IS6, *n* = 8; IS607, *n* = 8; and IS110, *n* = 6) were moderately represented, and the remaining 19 families were present only at minor frequencies. The distributions of the IS families and their members are summarized in Supporting Data S4. Altogether, the results showed that the genome of *C. tertium* HGMC01 contained numerous mobile genetic elements obtained from other bacteria or phages via HGT events, making it larger than the genomes of other *C. tertium* strains.

### 3.8 Virulence‐Associated Genes

Virulence factors in *C. tertium* HGMC01 were identified using bioinformatic approaches with PathogenFinder and manual search against a virulence factor database. According to PathogenFinder (Cosentino et al. 2013), *C. tertium* HGMC01 was classified as a nonhuman pathogen (0.469). Among five putative virulence factor proteins in the whole proteome, two proteins, C‐tertium_1_00485 and C‐tertium_1_01259, were matched to pathogenic protein families in *C. difficile* R20291 and *C. botulinum* Ba4 str. 657, respectively. Nevertheless, manual inspection of the annotated genome and BLAST searches against orthologous genes of other *C. tertium* strains identified four toxin‐coding genes and 10 other virulence factor‐coding genes (Table 5). Among these genes were *tab*A, encoding toxin‐antitoxin biofilm protein; *tel*A, encoding toxic anion resistance protein; *ndo*A, encoding endoribonuclease EndoA, and *tcd*A, encoding toxinA, each of which has been observed in other *C. tertium* strains (Muñoz et al. 2019). EndoA is a well‐known toxic component of a type II toxin‐antitoxin system, which can play a role in inhibiting protein synthesis by cleaving single‐stranded RNA. In addition, the *C. tertium* HGMC01 genome contained a gene encoding a hypothetical protein for Zeta toxin; however, other toxin‐related genes such as antitoxin HipB and its regulator gene *hip*B, which were found in the *C. tertium* Gcol_A2 strain, were not detected (Muñoz et al. 2019). Nevertheless, two genes encoding the HTH‐type transcriptional regulator *imm*R (C‐tertium_1_00793; C‐tertium_1_00794), which were previously found in *C. tertium* src5 (Muñoz et al. 2019), were also present in the *C. tertium* HGMC01 genome.

**Table 5:** Putative virulence‐associated genes in *C. tertium*.

Virulence factor	Locus tag	Genotype	Function
Toxin coding gene	C‐tertium_1_00172	*tab*A	Toxin‐antitoxin biofilm protein TabA
	C‐tertium_1_01263	*tox*Z	Zeta toxin
	C‐tertium_1_03099	*ndo*A	Endoribonuclease EndoA
	C‐tertium_1_03183	*tcd*A	ToxinA
Other virulence factors	C‐tertium_1_00181	*nan*H	Sialidase
	C‐tertium_1_00199	*nag*H1	Hyaluronoglucosaminidase 1
	C‐tertium_1_00574	*nag*H2	Hyaluronoglucosaminidase 2
	C‐tertium_1_00514	*fbp*	Fibronectin‐binding protein
	C‐tertium_1_01063	*fbp*	Putative protein YloA, Fibronectin‐binding A domain protein
	C‐tertium_1_01067	*eps*F1	Type II secretion system protein F
	C‐tertium_1_03793	*eps*F2	Type II secretion system protein F
	C‐tertium_1_00506	*tel*A	TelA‐like protein, Toxic anion resistance protein
	C‐tertium_1_02330	*cvf*B	Conserved virulence factor B
	C‐tertium_1_01092	*tly*A	Hemolysin A, 16S/23S rRNA (cytidine‐2’‐O)‐ methyltransferase TlyA

In addition to *tel*A, nine genes encoding other virulence factors were identified (Table 5). Among these, *nan*H, encoding sialidase; *fbp*, encoding fibronectin‐binding protein; and *tly*A, encoding hemolysin, have been commonly observed in several *Clostridium* strains (Grobe et al. 1998; Lewis and Lewis 2012; Kiu et al. 2017; Muñoz et al. 2019; Raimondi et al. 2021; Candeliere et al. 2024). Two genes in the *C. tertium* HGMC01 genome, *eps*F1 and *eps*F2, encoded type II secretion system protein F, which is known to deliver toxins and hydrolytic enzymes in both gram‐positive and gram‐negative bacteria for host infections (Korotkov and Sandkvist 2019). The *C. tertium* HGMC01 genome also contained two *nagH* genes encoding hyaluronoglucosaminidases, which are homologous to Mu‐toxin and have been found in some other Clostridial strains (Muñoz et al. 2019; Candeliere et al. 2024). These bacterial hyaluronidases are known as potential virulence factors in pathogenic Gram‐positive bacteria that initiate infections at skin or mucosal surfaces (Hynes and Walton 2000). Finally, the *C. tertium* HGMC01 genome contained *cvf*B encoding a conserved virulence factor B protein, which was previously reported to contribute to the expression of virulence factors such as hemolysin, DNase, protease, and protein A in the pathogenic bacterium *Staphylococcus aureus* (Kaito et al. 2005; Matsumoto et al. 2007).

### 3.9 Antibiotic‐ and Multidrug‐Resistance Genes

Antibiotic‐ and multidrug‐resistance genes in the *C. tertium* HGMC01 genome were identified using a manual search of CARD, which found a total of 163 candidate resistance genes (Supporting Data S5). Genes potentially conferring resistance against macrolides, fluoroquinolones, tetracyclines, glycopeptides, and peptide antibiotics accounted for 56% (92/163) of the candidates. Among these, there were 26 genes for resistance against macrolides [*mac*B, *mef*(B), *ole*C], 23 for fluoroquinolones (*gyr*A, *gyr*B, *pat*B, *abe*M, *arl*S, *cde*A), 15 for glycopeptide antibiotics (*van*S), 14 for tetracyclines [*tcr*3, *tet*(35), *tet*(T), *tet*A(58), *tet*B(60)], and 14 for peptide antibiotics (*arn*A, *bas*S, *cls*, *lps*B, *mpr*F, *ugd*). These genes were highly homologous to orthologs identified in pathogenic gram‐positive bacteria such as *Clostridioides difficile*, *Enterococcus faecium*, and *Streptococcus agalactiae* (Hernandez et al. 2021; Gu et al. 2022; Akter et al. 2023). In addition, the *C. tertium* HGMC01 genome contained genes with high homology to genes conferring resistance to aminocoumarin (*gyr*B, *nov*A), aminoglycosides (*kdp*D), isoniazid‐like antibiotic (*ndh*), lincosamides (*lmr*C, *lmr*D), nitroimidazoles (*msb*A), pleuromutilins (*tae*A), pyrazines (*rps*A), and β‐lactams antibiotics (*pdb*1, *pdb*2) in *Neisseria meningitidis* and *Helicobacter pylori*; rifamycins (*hel*R, *rpo*B) in *Mycobacterium tuberculosis*; phosphonic acid antibiotics (*mur*A) in *Borrelia burgdorferi* and *S. aureus*; and mupirocin‐like antibiotic (*mup*A, *mup*B) in *S. aureus*. Several genes, such as *efr*A, *cde*A, *optr*A, *vga*B, *cde*A, *arl*R, and *clb*B, that have been shown to confer multidrug resistance against 2–5 of the antibiotics mentioned above were also identified. In addition, putative genes associated with antibiotic modification/degradation, antibiotic efflux (exporter/binding), antibiotic target modification, and antibiotic target protection were also identified (Table 6). The presence of these genes in the *C. tertium* HGMC01 genome suggests that this strain is potentially a multidrug‐resistant strain.

**Table 6:** Putative bacteria multidrug resistance (MDR) associated genes in *C. tertium*.

Mechanism of MDR	Locus tag	Genotype	Function
Antibiotic			(β‐lactamase)
modification/degradation	C‐tertium_1_00340	*flp*	Protein flp, β‐lactamase
	C‐tertium_1_00581	*hyp*P	β‐lactamase domain protein
	C‐tertium_1_00847	*sme*1	Carbapenem‐hydrolyzing β‐lactamase
	C‐tertium_1_01189	*hyp*P	β‐lactamase
	C‐tertium_1_01190	*hyp*P	β‐lactamase
	C‐tertium_1_02784	*hyp*P	β‐lactamase
			(Modification/degradation)
	C‐tertium_1_00639	*glo*A1	Lactoylglutathione lyase
	C‐tertium_1_00647	*glo*A2	Lactoylglutathione lyase
	C‐tertium_1_00914	*glo*C	Hydroxyacylglutathione hydrolase GloC
	C‐tertium_1_01174	*pat*	Phosphinothricin *N*‐acetyltransferase
	C‐tertium_1_01175	*hyp*P	Aminoglycoside 6‐adenylyltransferase
	C‐tertium_1_01219	*ant*1	Streptomycin 3”‐adenyltransferase
	C‐tertium_1_01255	*stt*H	Streptothricin hydrolase
	C‐tertium_1_02223	*vat*	Virginiamycin A acetyltransferase VAT
	C‐tertium_1_02291	*hyp*P	Antibiotic biosynthesis monooxygenase
			(Resistance)
	C‐tertium_1_00642	*tet*M	Tetracycline resistance protein
	C‐tertium_1_00422	*mer*R1	Methicillin resistance mecR1 protein
	C‐tertium_1_00427	*van*W	Vancomycin B‐type resistance protein VanW
	C‐tertium_1_01767	*van*W	Vancomycin B‐type resistance protein VanW
	C‐tertium_1_02319	*van*W	Vancomycin B‐type resistance protein VanW
	C‐tertium_1_01460	*mar*A1	Multiple antibiotic resistance protein MarA1
	C‐tertium_1_02517	*mar*A2	Multiple antibiotic resistance protein MarA1
	C‐tertium_1_01191	*hyp*P	Glyoxalase/Bleomycin resistance protein
Antibiotic efflux			(ATP‐binding cassette superfamily)
(exporter/binding)	C‐tertium_1_01282	*bce*A1	Bacitracin export ATP‐binding protein BceA
	C‐tertium_1_01568	*bce*A2	Bacitracin export ATP‐binding protein BceA
	C‐tertium_1_02111	*bce*A3	Bacitracin export ATP‐binding protein BceA
	C‐tertium_1_02140	*bce*A4	Bacitracin export ATP‐binding protein BceA
	C‐tertium_1_01402	*drr*B	Daunorubicin/doxorubicin resistance ABC transporter permease protein DrrB
	C‐tertium_1_01403	*drr*A1	Daunorubicin/doxorubicin resistance ATP‐binding protein DrrA
	C‐tertium_1_01431	*drr*A2	Daunorubicin/doxorubicin resistance ATP‐binding protein DrrA
	C‐tertium_1_01567	*bce*B1	Bacitracin export permease protein BceB
	C‐tertium_1_02110	*bce*B2	Bacitracin export permease protein BceB
	C‐tertium_1_01464	‐‐‐	Fluoroquinolones export ATP‐binding protein
	C‐tertium_1_02619	*lag*D	Lactococcin‐G‐processing and transport ATP‐binding protein LagD
	C‐tertium_1_02257	‐‐‐	Putative multidrug export ATP‐binding/permease protein
	C‐tertium_1_02323	‐‐‐	Putative multidrug export ATP‐binding/permease protein
			(major facilitator superfamily)
	C‐tertium_1_01551	*mep*A1	Multidrug export protein MepA
	C‐tertium_1_01927	*mep*A2	Multidrug export protein MepA
	C‐tertium_1_02071	*mep*A3	Multidrug export protein MepA
	C‐tertium_1_02091	*mep*A4	Multidrug export protein MepA
	C‐tertium_1_02586	*mep*A5	Multidrug export protein MepA
	C‐tertium_1_02626	*mep*A6	Multidrug export protein MepA
	C‐tertium_1_02883	*mep*A7	Multidrug export protein MepA
	C‐tertium_1_03376	*mep*A8	Multidrug export protein MepA
	C‐tertium_1_02280	*bac*E	Putative bacilysin exporter BacE
Antibiotic target modification			(Penicillin‐binding protein)
	C‐tertium_1_00863	*pbp*F	Penicillin‐binding protein 1F
	C‐tertium_1_1705	*pbp*B	Penicillin‐binding protein B
	C‐tertium_1_3084	*pbp*A	Penicillin‐binding protein A
	C‐tertium_1_3479	*pbp*	β‐lactam‐inducible penicillin‐binding protein
Antibiotic target protection	C‐tertium_1_03335	*gyr*B	DNA gyrase subunit B (novobiocin)

### 3.10 Cell Wall‐Associated Components Causing Inflammatory Responses Via TLR2 and TLR4

**Figure 4:**
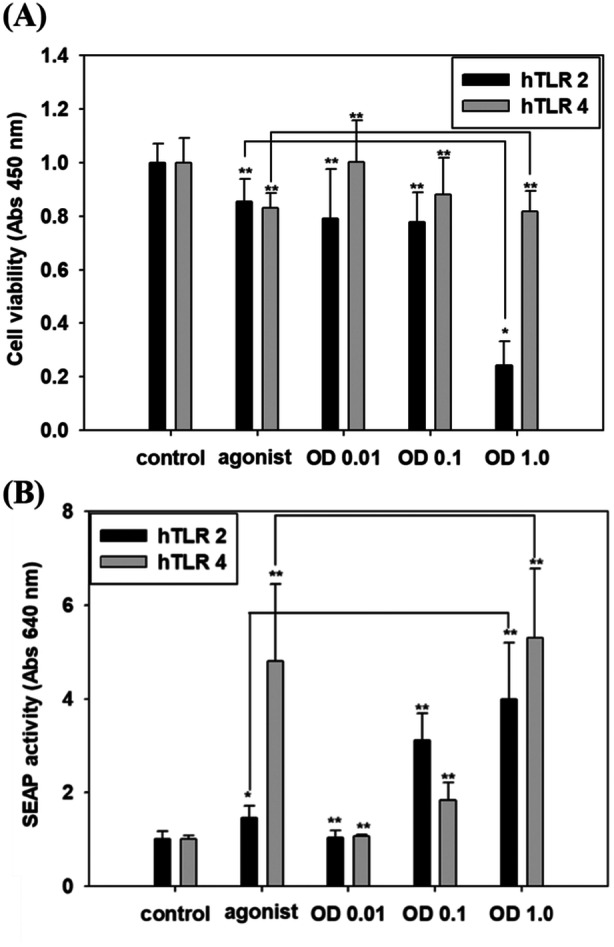
Cytotoxicity and relative stimulation of human toll‐like receptor 2 (hTLR2) and hTLR4 in HEK‐blue cells. (A) Viability of HEK‐blue cells expressing hTLR2 or hTLR4 was measured by cell counting kit‐8 after treatment for 16 h with three different concentrations (OD_600_ = 0.01, 0.1, or 1) of *Clostridium tertium* HGMC01 fixed whole cells. The agonists Pam_3_CSK_4_ (5 ng/mL) for hTLR2 and lipopolysaccharide (*Escherichia coli* O55B5, 50 ng/mL) for hTLR4 were used as positive controls. (B) hTLR2/hTLR4 stimulation activities were detected as secreted embryonic alkaline phosphatase (SEAP) after treatment under the same conditions as in (A). Data are presented as mean ± SD of six independent replicates (*n* = 6). *p < 0.05; **p < 0.01.

The potential for cell wall‐associated components to cause cytotoxicity and immune stimulation was analyzed using HEK‐blue cells transfected with hTLR2 or hTLR4 and an inducible SEAP reporter gene. Cytotoxicity was detected by cell counting kit‐8 assay. Fixed whole cells of *C. tertium* HGMC01 induced cytotoxicity in a dose‐dependent manner in both TLR‐expressing HEK‐blue cell lines, although the cytotoxic effect was stronger against HEK‐blue hTLR2 cells than against HEK‐blue hTLR4 cells (Figure 4A).

Fixed whole cells of *C. tertium* HGMC01 induced SEAP activity by modulating hTLR2 or hTLR4 activity in a dose‐dependent manner (Figure 4B). At a low concentration of *C. tertium* HGMC01 cells (0.01 OD_600_), the TLR2/4 activity levels were not different than those of negative controls with untreated cells. However, increased concentrations of *C. tertium* HGMC01 cells dramatically enhanced the TLR2/4 activities. The activity of hTLR2 was relatively higher than that of hTLR4, as the gram‐positive cell wall components of *C. tertium* HGMC01 could stimulate hTLR2 activation. Nevertheless, the whole‐cell treatment also increased hTLR4 activity similarly to LPS, which is a major outer‐membrane component of Gram‐negative bacteria and was used as a positive control. These results revealed that the cell wall components of *C. tertium* HGMC01 can modulate TLR2‐ and TLR4‐dependent signaling pathways in human cells.

**Figure 5:**
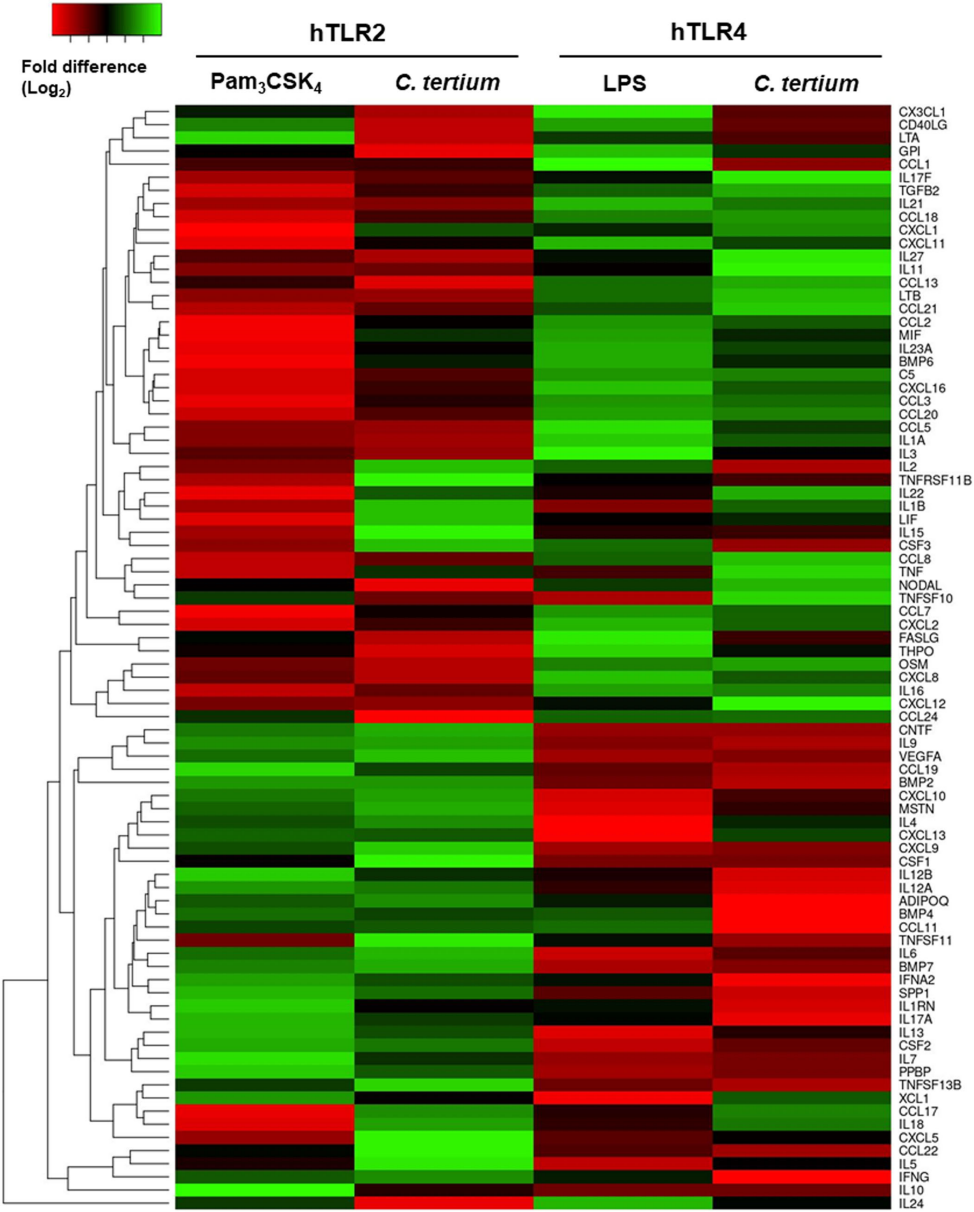
Transcriptional responses of cytokine and chemokine genes in HEK‐blue cells in response to stimulation with *Clostridium tertium* HGMC01 fixed whole cells. The gene expression heat map displays the transcriptional responses (compared with untreated cells) of HEK‐blue cells expressing hTLR2 or hTLR4 after treatment with *C. tertium* cells or the TLR agonists Pam_3_CSK_4_ or LPS.

To further analyze the effects of the *C. tertium* HGMC01 cell wall components on host inflammatory response, the transcriptional responses in HEK‐blue hTLR2 or hTLR4 cells were analyzed by qPCR using primers specific for 84 cytokines and chemokines. Positive controls for HEK‐blue hTLR2 or hTLR4 cells were treated with the TLR2 agonist Pam_3_CSK_4_ or the TLR4 agonist LPS, respectively. Treatment with *C. tertium* HGMC01 cells stimulated hTLR2 and hTLR4 activities and increased the expression of 53 cytokine/chemokine genes in HEK‐blue hTLR2 cells and 60 cytokine/chemokine genes in HEK‐blue hTLR4 cells (Figure 5). In addition, 36 cytokine/chemokine genes were upregulated by treatment with *C. tertium* HGMC01 cells in both HEK‐blue cell lines compared with nontreated controls. In the HEK‐blue hTLR2 cells, 39 genes were commonly upregulated by *C. tertium* HGMC01 cells and Pam_3_CSK_4_. Similarly, in the HEK‐blue hTLR4 cells, 47 genes were commonly upregulated by *C. tertium* HGMC01 cells and LPS. Overall, the *C. tertium* HGMC01 cells stimulated cytokine/chemokine gene expression more strongly in hTLR4‐expressing cells than in hTLR2‐expressing cells. Nevertheless, these data revealed that the cell wall components of *C. tertium* HGMC01 are potential virulence factors causing a TLR2/TLR4‐associated transcriptional response.

Whole *C. tertium* HGMC01 cells induced comparable changes to regulated gene expression in hTLR2‐expressing and hTLR4‐expressing HEK‐blue cells, which were distinct from those induced by Pam_3_CSK_4_ and LPS. These patterns were dependent on the hTLR2 or hTLR4 expression in each cell line (Figure 5). Although the expression levels were slightly different, the cytokines interleukin (IL)‐6, IL‐9, IL‐12A, IL‐12B, IL‐18, IL‐24, and IL‐27 and the chemokines CXCL5, CXCL8, CCL17, and CCL19 were commonly stimulated in all the tested cells. Interestingly, the cytokines IL‐1β, IL‐2, IL‐4, IL‐5, IL‐6, IL‐7, IL‐9, IL‐10, IL‐12A, IL‐12B, IL‐13, IL‐15, IL‐17A, IL‐18, and IFN‐γ and the chemokines CXCL9, CXCL10, CXCL13, CXCL17, CCL11, CCL19, CCL17, and CCL22, which are known to be stimulated by bacterial infection, were highly upregulated in HEK‐blue hTLR2 cells stimulated with *C. tertium* HGMC01 cells. In contrast, the cytokines IL‐1α, IL‐11, IL‐16, IL‐17F, IL‐21, IL‐23A, IL‐27, and TNF‐α and the chemokines CXCL1, CXCL2, CXCL8, CXCL11, CXCL12, CXCL16, CCL2, CCL3, CCL5, CCL7, CCL8, CCL13, CCL18, CCL20, CCL2, and CCL24 were relatively highly expressed in HEK‐blue hTLR4 cells after exposure to whole *C. tertium* HGMC01 cells. These results indicate that the cell wall components of *C. tertium* HGMC01 played a role in triggering the induction of various cytokines and chemokines for immune response and inflammatory reaction.

## 4 Discussions

*C. tertium* can cause bacteremia in neutropenic and nonneutropenic patients and displays resistance against a broad spectrum of β‐lactam and lincosamide antibiotics (Thaler et al. 1986; Johnson and Tenover 1988; Rampling 1988; Alvarado‐Rodríguez and Quesada‐Gómez 2025). Although *C. tertium* was once regarded as a nonpathogenic bacterium, its ability to cause diverse pathologies, such as enterocolitis, bacteremia, septicemia, antibiotic resistance, gastrointestinal disease, and/or neutropenia, suggest that it should be regarded as an emerging pathogen (Coleman et al. 1993; Steyaert et al. 1999; Miller et al. 2001; Tappe et al. 2005; Bonda et al. 2022; Justesen et al. 2022; Kim et al. 2023; Alvarado‐Rodríguez and Quesada‐Gómez 2025). The pathogenicity of *C. tertium* varies among different diseases and patient cases (Miller et al. 2001; Tappe et al. 2005; Bonda et al. 2022; Justesen et al. 2022; Kim et al. 2023), reflecting the genetic diversity found in clinically isolated *C. tertium* strains.

*C. tertium* HGMC01, isolated by enrichment culture of human fecal matter, showed a larger genome size compared with orthologous *C. tertium* strains (Table 1). Although the average genome size of *C. tertium* strains is 3877 ± 70 kb, the genome of *C. tertium* HGM01 is approximately 100–200 kb larger. In addition, a pangenome analysis revealed that the *C. tertium* HGMC01 genome contained the highest number of unique genes and the lowest number of accessory genes among the orthologous genomes. Although *C. tertium* HGMC01 was isolated from a human source, a phylogenic analysis based on a core *C. tertium* genome indicated that it is closely related to *C. tertium* src5, which is of animal origin (Muñoz et al. 2019). The genomic variation and phylogenetic relationships within the same species suggest that *C. tertium* HGMC01 gained extra genes from various bacteria and viruses by HGT facilitated by diverse mobile genetic elements. Thus, *C. tertium* HGMC01, like other Gram‐positive pathogens, came to harbor distinct features for pathogenicity via rapid evolution and adaptation in gut niches (Broaders et al. 2013; Juhas 2015; Udaondo et al. 2022).

Although the ability to produce various toxins varies among *C. tertium* strains, the *C. tertium* HGMC01 genome harbored three toxin‐related genes (Table 5). Comparison to previously identified *C. tertium* strains showed that the *C. tertium* HGMC01 genome contained *tcd*A, encoding *C. difficile* toxin A, but not *tcd*B or *tox*B, similar to the highly cytotoxic *C. difficile* Gcol.A43 (Muñoz et al. 2019). Interestingly, the *C. tertium* HGMC01 genome also contained a Zeta toxin gene (*tox*Z), which was previously found in *Clostridium paraputrificum* strains but not in *C. tertium* (Muñoz et al. 2019). In addition, the *C. tertium* HGMC01 genome contained TabA, encoding a toxin‐antitoxin biofilm protein. Although TabA is known to play roles in the inducible toxicity of type I toxins, stress response, biofilm structure, and genomic region stabilization in *C. difficile*, its biological functions as a virulence factor remain to be unveiled in *C. tertium* and other pathogenic bacteria (Soutourina 2019; Singh et al. 2021). Compared with other Clostridial strains, *C. tertium* HGMC01 displayed relatively high frequencies of other virulence factors including sialidase (*nan*H), hyaluronidase (a mutoxin, *nag*H1 and *nag*H2), fibronectin‐binding protein (*fbp*A/B), Type II secretion system protein (*esp*F), endoribonuclease (*ndo*A), conserved virulence factor B (*cvf*B), and hemolysin proteins (Muñoz et al. 2019; Candeliere et al. 2024). Recently, the comparative genome analysis also revealed that *C. tertium* shared multiple homologous pathogenicity‐associated virulence factor proteins with other known pathogenic *Clostridium* species (Qing et al. 2025). These data also showed that all virulence factors of *C. perfringens* annotated in *C. tertium* genome, suggesting that horizon gene transfer (HGT) may occur quite often between the two species. In our study, the presence of various virulence factors suggests that the pathogenicity of *C. tertium* can be expanded depending on the presence of specific virulence factors, providing distinguished characteristics compared with other variants of the same species.

Moreover, CAZyme analysis and gene annotation for sialic acid catabolism indicated that *C. tertium* HGMC01 can hydrolyze mucin‐type O‐glycans and metabolize their monosaccharides (Tables 3 and 4). The presence of genes for various secreted glycosidases, including (sialidase, β‐1,3(4)‐galactosidase, exo‐α‐*N*‐acetyl galactosaminidase, β‐hexosaminidase, *N*‐acetyl‐β‐glucosaminidase, α‐*N*‐acetylglucosaminidase, and glycopeptide α‐*N*‐acetylgalactosaminidase), implies that *C. tertium* HGMC01 can remove sugars in core and extended O‐glycan structures to obtain nutrients in host gut niches (Figure 3). The *C. tertium* HGMC01 genome did not contain any fucosidase genes; however, the fucose moiety linked to core type O‐glycans might be eliminated by fucosidases secreted by other mucus‐associated gut microbes such as *Bacteroides*, *Bifidobacterium*, and *Ruminococcus* species (Singh et al. 2021). A previous study reported that mucin‐degrading *C. tertium* WC0709 can utilize galactose, *N*‐acetylglucosamine, *N*‐acetylgalactosamine, and other monosaccharides, except for D/L‐fucose (Candeliere et al. 2024). For mucin glycan catabolism, *C. tertium* WC0709 has genes encoding the sialic acid transporter NanT and other sugar transporters. *C. tertium* WC0709 lacks genes for the biosynthesis of sialic acid (neuAB) or nonulosinuc acid (nul) (Yamaguchi and Yamamoto 2023), suggesting that it can take up and utilize host sialic acid glycans without endogenous sialic acid biosynthesis. In contrast to *C. tertium* WC0709, which lacks a *nan*A gene (Candeliere et al. 2024), *C. tertium* HGMC01 has all the genes needed for sialic acid catabolism within upstream (*nan*A/E/K) and downstream (*nag*A/B) pathways. Although many bacteria have a cluster (Nan cluster) of core genes encoding N‐acetyl neuraminic acid lyase (NanA), epimerase (NanE), and kinase (NanK) (Yamaguchi and Yamamoto 2023), the catabolic sialic acid genes are not clustered in *C. tertium* HGMC01 but are instead distributed across the entire genome (Table 4). Furthermore, multiple isoenzymes of *nan*A and *nan*E for upstream sialic acid catabolism and *nag*A for downstream sialic acid catabolism were also observed in *C. tertium* HGMC01. Although our pangenome analysis showed that all the genes for sialic acid catabolism, except for *nan*T, were present in all six *C. tertium* strains (Supporting Data S1), it is still unknown whether these are endogenous or exogenous genes for the bacterial pathogenicity. Further studies are needed to evaluate the evolution of genes and gene clusters for sialic acid catabolism in pathogenic *Clostridium* species by HGT from other gut microbes.

In addition to hydrolyzing mucin O‐glycans, a variety of glycosidases in *C. tertium* HGMC01 and other Clostridial strains may promote virulence by de‐glycosylating host cell‐surface glyconjugates to enhance host‐bacteria interaction, adhesion, or invasion (Yamaguchi and Yamamoto 2023). It is still unknown whether *C. tertium* HGMC01 can adhere to specific glycan structures on the surface of host cells. Nevertheless, a secreted protein (C‐tertium_1_03740) consisting of three CBM51 and one CBM32 for blood group A/B‐antigen that was predicted to bind galactose was commonly present in the genomes of *C. tertium* HGMC01 and the other five ortholog strains (Table 3 and Supporting Data S1). The human gastric mucin O‐glycans harbor blood group A/B‐antigens, providing glycan structures that can serve as potential binding sites for pathogenic bacteria (Kappler and Hennet 2020; Jajosky et al. 2022). As a possible virulence factor, the presence of this protein in *C. tertium* suggests that *C. tertium* strains can recognize blood group antigens as well as mucin O‐glycans as glycan epitopes by acting as initial attachment factors and specific entry receptors on host cell surfaces.

No *C. tertium* strain has yet been found to cause inflammatory bowel disease. Nonetheless, several cases of colitis caused by *C. tertium* strains have been reported (Chalhoub et al. 2016; Li et al. 2021; Tanwar et al. 2023). Depending on the diversity of genes encoding toxins, virulence factors, and enzymes for mucin O‐glycan degradation, *C. tertium* strains, like *C. difficile* strains, may gain different virulence capabilities and then affect inflammatory bowel disease (Labourel et al. 2023; Tanwar et al. 2023). Our results demonstrated that *C. tertium* HGMC01 can contribute to cytotoxicity and TLR‐mediated host inflammatory response (Figures 4 and 5). The whole‐cell treatments stimulated both hTLR2 and hTLR4. The cell wall of *C. tertium* HGMC01 may contain various glycoconjugates known as TLR2 ligands, including peptidoglycan (PG), lipoteichoic acid (LTA), lipopeptide/protein (LP), capsular polysaccharides (CPs), and exopolysaccharides (EXs) (Muzio et al. 2000). Although TLR4 is mainly activated by LPS‐mediated immune responses to Gram‐negative bacteria, it can also respond to a wide variety of bacterial glycoconjugates, endotoxic LPS partial structures, and gram‐positive LTA, but not to gram‐positive bacteria, PG, or LP (Dziarski and Gupta 2000). Therefore, the LTA structures in the cell wall of *C. tertium* HGMC01 may have contributed to the hTLR4‐dependent response.

In contrast to the extensive data for other pathogenic bacteria, the TLR‐mediated signaling response toward *Clostridium* species has been studied only in *C. perfringens* and *C. difficile* (Shi et al. 2019; Lai et al. 2021). Infections with these two species in animal models induced immune responses involving the TLR2 or TLR4 signaling pathways, which enable pathogen sensing for innate immunity. Like *C. perfringens* and *C. difficile*, *C. tertium* HGMC01 induced upregulation of a variety of cytokine‐ and chemokine‐related genes. Additionally, a clinical report revealed that infection with Clostridiaceae upregulated TLR2/4 and resulted in bacterial colonization in patients with chronic/relapsing pouchitis (Scarpa et al. 2011). These results also support the hypothesis that infection with *C. tertium* and other species depends on interactions between bacterial cell wall components and TLR‐mediated signaling in host cells. However, it is still unknown which glycoconjugates in the cell wall of *C. tertium* HGMC01 can trigger TLR signaling in host cells. Recently, cell wall glycopolymers consisting of a branched, hexasaccharide repeating unit containing phosphate as a conserved antigen were identified in various *C. difficile* strains (Chu et al. 2016). Although this glycoconjugate was not tested for its ability to induce TLR2/4 expression, the core antigen structure, which contains hexasaccharide consisting of three monosaccharides, glucose, *N*‐acetylgalactosamine, and mannose with phosphate, is a potential antagonist for inducing TLR‐mediated signaling. *C. tertium* HGMC01 may synthesize similar glycoconjugates because its genome contains several putative loci for the biosynthesis of polysaccharides, such as PG, LTA, CPs, and EXs. Further studies will aim to identify its glycan structures and virulence activity toward host cells.

*C. tertium* strains harbor resistance toward various β‐lactam and lincosamide antibiotics (Thaler et al. 1986; Johnson and Tenover 1988; Rampling 1988; Alvarado‐Rodríguez and Quesada‐Gómez 2025). Recently, clinically isolated strains increased their antimicrobial resistances against vancomycin and clindamycin (Alvarado‐Rodríguez and Quesada‐Gómez 2025). Furthermore, previous studies showed that *C. tertium* WC0709 was resistant to tetracyclines, chloramphenicol, the aminoglycoside gentamicin, and the β‐lactam antibiotics ampicillin and penicillin G (Candeliere et al. 2024). Our genome analysis provides some genetic clues about why clinically isolated *C. tertium* strains have antibiotic and multidrug resistance (Table 6). For antibiotic modification or degradation, various hydrolases and modification enzymes can contribute detoxification of β‐lactam, phosphonic, glycopeptide, and aminoglycoside antibiotics, as well as aziridine‐containing drugs. In addition, several antibiotic resistances and target‐binding proteins such as penicillin‐binding proteins against β‐lactam antibiotics can provide enhanced antibiotic resistance. Moreover, a variety of multidrug export proteins and other drug permeases in *C. tertium* HGMC01 can also confer resistance to a broad spectrum of antibiotics. Interestingly, specific exporters of antimicrobial peptides such as bacitracin and lactococcin‐G bacteriocin among antibiotic efflux‐related protein groups can eliminate toxic compounds to protect the bacterium and enable it to survive within a diverse microbial community. Moreover, a number of mobile genetic elements in *C. tertium* HGMC01 may spread antibiotic resistance ability to other *Clostridioides* genus by HGT. Although these mechanisms need to be confirmed by further studies, the diverse genes associated with multidrug resistance in *C. tertium* HGMC01 indicate that this strain should be considered a potentially high‐risk bacterium with the potential to cause clinical disease.

## 5 Conclusion

*C. tertium* HGMC01 isolated from human fecal matter displayed pathogenic potential with a large genome size and high numbers of mobile genetic elements, virulence factors, and antibiotic/multidrug‐resistant genes. Comprehensive genome analysis with five other *Clostridium* strains identified evolutionary relationships and differences in genome characteristics. A detailed analysis of genes encoding CAZymes and enzymes for sialic acid catabolism suggested that this strain interacts with host cells by degrading mucin in the gut, potentially through recognition or adhesion to glycoconjugates of mucin‐type O‐glycans. Cytotoxic effects and TLR 2/4‐mediated immune response in host cells suggest that glycoconjugates in the cell wall of *C. tertium* HGMC01 are additional virulence factors. Further studies are needed to confirm virulence factors and antibiotic/multidrug resistances in this strain; however, our results provide clues about how Clostridial strains enhance pathogenicity by gaining virulence factor‐related genes through HGT facilitated by a variety of mobile genetic elements.

## Author Contributions

**Seonghun Kim:** conceptualization, investigation, funding acquisition, writing – original draft, writing – review and editing, visualization, validation, software, formal analysis, project administration, data curation, supervision, resources. **Ji Young Kang:** data curation, writing – review and editing, validation. **Jung‐Sook Lee:** resources, data curation, funding acquisition.

## Ethics Statement

The authors have nothing to report.

## Conflicts of Interest

The authors declare no conflicts of interest.

## Supporting information

Supporting data 1. Pangenome analysis data.

Supporting data 2. MobileOG alignment data.

Supporting data 3. Prophage analysis data.

Supporting data 4. IS analysis data.

Supporting data 5. CARD analysis data.

## Data Availability

The authors confirm that the data supporting the findings of this study are available within the article and its Supporting materials. Raw data deposit: https://doi.org/10.6084/m9.figshare.27645603.v2 Genome data deposit (NCBI): https://www.ncbi.nlm.nih.gov/bioproject/PRJNA1180257.
